# Weighted Kernel Filter Based Anti-Air Object Tracking for Thermal Infrared Systems

**DOI:** 10.3390/s20154081

**Published:** 2020-07-22

**Authors:** Chuljoong Kim, Hanseok Ko

**Affiliations:** 1 Department of Video Information Processing, Korea University, Seoul 136-713, Korea; cjkim@ispl.korea.ac.kr; 2 Hanwha Systems Co., Sungnam 461-140, Korea

**Keywords:** visual object tracking, thermal infrared (TIR), region proposal network (RPN), convolutional neural network (CNN), weighted kernel filter (WKF)

## Abstract

Visual object tracking is an important component of surveillance systems and many high-performance methods have been developed. However, these tracking methods tend to be optimized for the Red/Green/Blue (RGB) domain and are thus not suitable for use with the infrared (IR) domain. To overcome this disadvantage, many researchers have constructed datasets for IR analysis, including those developed for The Thermal Infrared Visual Object Tracking (VOT-TIR) challenges. As a consequence, many state-of-the-art trackers for the IR domain have been proposed, but there remains a need for reliable IR-based trackers for anti-air surveillance systems, including the construction of a new IR dataset for this purpose. In this paper, we collect various anti-air thermal-wave IR (TIR) images from an electro-optical surveillance system to create a new dataset. We also present a framework based on an end-to-end convolutional neural network that learns object tracking in the IR domain for anti-air targets such as unmanned aerial vehicles (UAVs) and drones. More specifically, we adopt a Siamese network for feature extraction and three region proposal networks for the classification and regression branches. In the inference phase, the proposed network is formulated as a detection-by-tracking method, and kernel filters for the template branch that are continuously updated for every frame are introduced. The proposed network is able to learn robust structural information for the targets during offline training, and the kernel filters can robustly track the targets, demonstrating enhanced performance. Experimental results from the new IR dataset reveal that the proposed method achieves outstanding performance, with a real-time processing speed of 40 frames per second.

## 1. Introduction

Visual tracking has received significant research attention in recent years, with infrared (IR) object tracking in particular important for military uses [1], such as surveillance and missile and laser weapon systems. IR radiation is composed of electromagnetic radiation that has a longer wavelength than visible radiation. It is divided into near-wave IR (NIR, 700–1000 nm), short-wave IR (SWIR, 1000–3000 nm), middle-wave IR (MWIR, 3000–5000 nm), and long-wave IR (LWIR, 7500–12,000 nm). Normally, MWIR and LWIR are referred to as thermal IR (TIR).

NIR and SWIR have a disadvantage in that they are not clearly visible at night. On the other hand, TIR is more commonly used to observe any object because it operates in the region of the spectrum where the thermal contrast is much higher due to black body physics. This allows TIR to be used in general visual object tracking for grayscale sequences [2]. As a consequence, any effective general image tracker, such as those using RGB images, is capable of TIR tracking, and this has led to the development of many TIR applications, including pedestrian detection and object tracking [3].

However, some key differences between RGB and TIR trackers mean that the use of an TIR tracker is much more difficult. The most obvious difference is that color information, a fundamental feature of targets in RGB images, is absent in the TIR domain, meaning that trackers that depend heavily on color information may not be able to track in the TIR domain. For instance, two objects with different color information but similar temperature distributions cannot be distinguished using TIR, confusing the tracker. Another difference is that the intensity of the objects in TIR images is represented by their temperature distribution rather than their illumination. Therefore, objects in TIR images are more likely to merge with the background.

The discriminative correlation filter (DCF) trains the ridge regressor via circular correlation and operates in the Fourier domain. trackers using DCF can conduct online tracking and update the weight of the filter at the same time. Many DCF based trackers have been widely used in the tracking community [4,5,6,7], and correlation-filter-based trackers that extract deep features to improve accuracy have recently been introduced [8,9,10]. Convolutional neural network (CNN)-based trackers use various deep features and have an outstanding tracking performance [11,12,13,14,15] due to their strong feature learning abilities. However, for TIR input, these trackers are not sufficiently effective. For instance, in the VOT-TIR 2017 challenge, ECO that is trained on the RGB domain ranked fifth out of ten trackers [16].

Due to the VOT-TIR challenge, various trackers that have been optimized for the IR domain have been introduced and tracking performance has greatly improved for IR images. In the VOT-TIR challenges, its objective is mainly to track for ground targets such as pedestrian, car, and bike which are clearly classified. In addition, there is an attempt to develop an algorithm to track various targets from videos that is captured by UAV [17]. Nevertheless, it is a formidable task develop trackers which tracks various size of drones and UAVs approaching from the sky. Moreover, there have been no attempts to create an anti-air dataset. For these reasons, in order to solve the limitations with the existing trackers and improve the anti-air tracking performance, we are about to develop a tracker for anti-air TIR images in this study.

To achieve this, a new dataset of anti-air TIR images is created and a robust anti-air TIR object tracking method with an end-to-end convolutional neural network (CNN) framework is proposed. In addition, a preprocessing procedure for the raw TIR images is developed because all TIR cameras have various bit-depths, so they must be normalized. Finally, a simple image enhancement method is utilized to reduce the computational time.

In terms of the network architecture for the proposed method, feature extraction is first trained using a modified version of MobileNetV2 [18] as the backbone network. Following this, a Siamese structure with region proposal networks (RPNs) is employed. Inspired by standard RPNs [19], the RPNs are modified to utilize a cross-correlation feature map of Siamese branches. For the cross-correlation, a layer-wise operation method is adopted, enabling the tracker to predict the similarity map from features learned at multiple levels. Because the similarity map is produced using cross-correlation at multiple levels, a depth-wise cross-correlation structure is used, which greatly reduces the number of parameters and the computational time.

For inference, the weighted kernel filters are employed in the template branch as a local detection task after the bounding box in the first frame is derived. The kernel filters are updated for every frame because they contain information from the past to the present. Lastly, after cross-correlation, the RPNs are summed with weights to predict the size and the position of the target.

The main contributions of this study are as follows:

(1) We create a new dataset for anti-air TIR images and propose an image-enhancement method for anti-air targets.
(2) We propose a Siamese architecture with a modified backbone and fused RPNs. This network is fully trained end-to-end without using pre-trained parameters for the RGB domain.
(3) In the inference phase, we utilize the weighted kernel filters that are updated for every frame.

Based on the contributions above, we develop a state-of-the-art visual tracking model that improves tracking accuracy. Separate training, validation, and test datasets are created from the anti-air TIR dataset for use with the tracker. The tracker is implemented in Python using PyTorch libraries.

## 2. Related Work

This section briefly summarizes the development of common datasets, recent trackers, and deep architectures.

### 2.1. Datasets

Quality datasets are crucial for vision-based computer applications, particularly in the training and testing of algorithms. ImageNet [20] is a dataset containing more than 14 million images, while YouTube-Bounding Boxes (YouTube-BB) [21] is a large-scale dataset of video URLs with densely-sampled high-quality single-object bounding box annotations. COCO [22] is a large-scale dataset used for the detection and the precise localization of objects, and the VOT and MOT Challenges produce new datasets annually. These datasets are mainly created to overcome various common difficulties for visual object tracking. As a consequence, by collecting a diverse range of image data, they have enabled the rapid development of object tracking and detection.

Though these datasets target RGB images, there have been attempts to create IR datasets. In the VOT 2019 challenge, a TIR dataset was introduced for IR trackers. VOT-TIR2016 [23] has 25 thermal infrared sequences and each sequence has six local attributes which can be used to test the performance of the tracking algorithm on frames with specific attribute evaluation. PTB-TIR [24] includes a number of thermal infrared video dataset for pedestrian tracking. RGB-T [25] includes 234 comprehensive thermal video dataset and takes into consideration of various environmental challenges. However, none of the data target the anti-air target dataset. Therefore, the creation of an anti-air IR dataset for electro-optical systems is required.

### 2.2. Trackers

The accuracy, efficiency, and robustness of the visual object tracking have rapidly improved since MOSSE [26] was first introduced. MOSSE was introduced to transform the template-matching problem into a correlation operation in the frequency domain. Due to this transformation, many trackers based on correlation filters are able to achieve efficient operating speeds and higher accuracy if the features are extracted effectively [4,5,6,7,27]. ECO-TIR [28] utilizes an end-to-end network to generate synthetic thermal data from RGB sequences and this could overcome the need of alignment of RGB and thermal data. MMNet [29] proposed a multi-task framework to learn the TIR-specific discriminative features and fine-grained correlation features for TIR tracking.

Siamese-based trackers have received significant attention for their accuracy and efficiency [9,11,14,15,30,31,32]. These trackers consist of two branches that encode input patches to another space and then join them as a cross-correlation operation to produce a similarity map. Inspired by the template-matching method, Siamese-FC [11] introduced a cross-correlation layer in a Siamese network without online updating and achieved high accuracy and speed. MCFTS [31] used pre-trained VGG-Net Siamese network for thermal infrared and proposed a correlation filter based ensemble method. It also proposed a fusion method based Kullback–Leibler divergence. CFnet [30] improved on the Siamese network-based tracking framework by adding a correlation filter to a Siamese network, making it shallower but more efficient. DCFNet [9] proposed a similar Siamese network that was trainable online by replacing the correlation layer was a discriminative correlation filter and that performed offline training through the Siamese network. HSSNet [32] introduced several multiple feature fusion based methods to overcome the limitations of one single feature in visual object tracking. In addition, state-of-the-art trackers [14,15] have introduced RPNs as a detection task after the Siamese network and have produced very promising results. However, none of these trackers have been applied to the anti-air TIR domain, thus their tracking accuracy needs to be validated in this respect.

### 2.3. Deep Architectures

Research on network architecture has rapidly developed since AlexNet [33] was first introduced in 2012, and many complex deep network architectures have since been proposed, such as VGGNet [34], GooLeNet [35], ResNet [36], MobileNet [37], and MobileNetV2 [18]. These architectures can not only be trained well with deeper structures, but have also greatly assisted the development of computer vision applications, such as object detection [38] and image segmentation [39]. For VOT, many deeper architectures have been adopted, producing promising results. SiamMask [40] introduced a rotational bounding box as a binary mask and classified pixel-wise belongingness for the target. For feature extraction, it adopted resnet-50, which is deeper network architecture. D&T [41] simultaneously produced regression-based tracking boxes and detection boxes with resnet-101 for feature extraction, and the detection boxes were linked and re-scored based on the tracking boxes. However, none of these architectures have been trained on anti-air TIR images. Therefore, this study looks to train all of the parameters within the entire network in the TIR domain.

## 3. Proposed Method

This section describes the development of the anti-air TIR dataset and the proposed Siamese-based network architecture with RPNs. In addition, the tracking process using cumulated kernel filters is explained.

### 3.1. Anti-Air TIR Dataset

#### 3.1.1. Data Collection

In order to produce an anti-air TIR dataset, object categories, such as drones, UAVs, and missiles, must be identified. The defense industry is most interested in systems that can track small anti-air objects such as drones and UAVs, thus an anti-air dataset needs to be created for deep-learning-based trackers. Because cameras in electro-optical systems must also be able to observe their surroundings during both the day and at night, TIR images are the most suitable choice for this dataset. Moreover, in order to collect anti-air IR data with various size, we need a camera to be able to observe small objects from far distances. For instance, if we need to observe 30 cm objects such as small drones within 2 km with 480 × 480 camera resolutions, we need a camera with less than a 1-degree field of view (FOV) because it is observed at 4 × 4 pixels. Therefore, we use a military-use TIR camera developed by Hanwha Systems and it’s FOV is 1-degree with 480 × 480 resolution.

**Figure 1:**
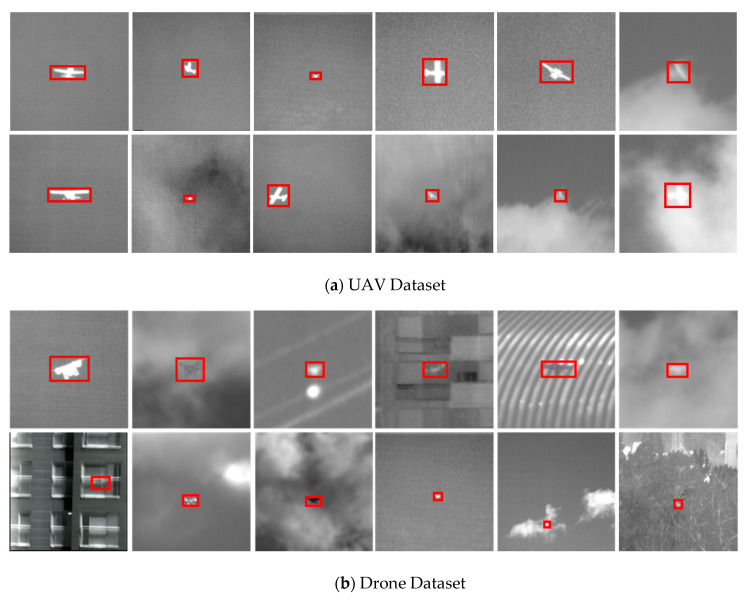
Proposed anti-air TIR dataset: (**a**) UAVs collected when the shape changes with a cloudy or sky background and (**b**) drones captured at low altitudes.

Figure 1. presents images of small drones and UAVs from the proposed TIR dataset. To ensure a variety of data, if at least one of the shape, scale, background, or pixel intensity of the target changes, data extraction is attempted. However, to prevent overfitting, data with similar shapes or little change in the background are excluded. The accurate tracking of drones has two major considerations. First, drones are typically symmetrical, thus data should be acquired when the angle of the drone changes. Second, the pixel intensity of a drone is sometimes darker than its surroundings if it has a lower temperature distribution than the background. When this happens, data should be extracted. In addition, almost all of the background data are collected at low altitudes and thus often include buildings, forests, or mountains. For UAVs, the data is collected from various angles (e.g., left-to-right, right-to-left, or rotation) and the background data are generally acquired at high altitudes, such as flying in front of clouds. The overall dataset is separated into training with 300 sequences and 78,903 frames, validation with 70 sequences and 21,046 frames, and test with 30 sequences and 14,750 frames.

#### 3.1.2. Preprocessing

In electro-optical systems, most TIR cameras do not have the same data structure. Therefore, the data must be converted to a certain bit-depth, such as RGB channels. To do this, how best to change the data needs to be decided. This is not a problem for high-temperature objects, which can be normalized within 0–255 with the min/max values of the current frame. In other words, they can be tracked robustly despite changes in illumination. However, for low-temperature objects, such as drones and UAVs, if the images are normalized using this technique, the pixel distribution fluctuates dramatically between frames due to the high variation in the pixel intensity. For this reason, we propose a preprocessing procedure that matches the bit-depths to 8 bits, improves image quality, and leads to less variation in the pixel intensity.

(1) $$mean_{new}=\frac{1}{N}\overset{N-1}{\underset{i=0}{\sum }}M_{i},\;\;std_{new}=\frac{1}{N}\overset{N-1}{\underset{i=0}{\sum }}S_{i}$$

(2) $$\begin{array}{c} \min=mean_{new}-4\times std_{new}\\ \max=mean_{new}+4\times std_{new} \end{array}$$

(3) $$Image_{new}=\frac{Image_{raw}-\min}{\max-\min}\times 255$$

**Figure 2:**
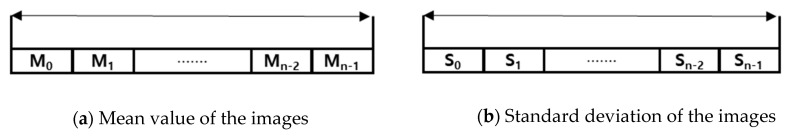
The structure of the circular buffer: (**a**) mean and (**b**) standard deviation of the images. All values are updated based on the frame number index. For instance, assuming that the buffer consists of 50 frames and the current frame number is 138, the buffer index is 138 mod 50 = 38.

**Figure 3:**
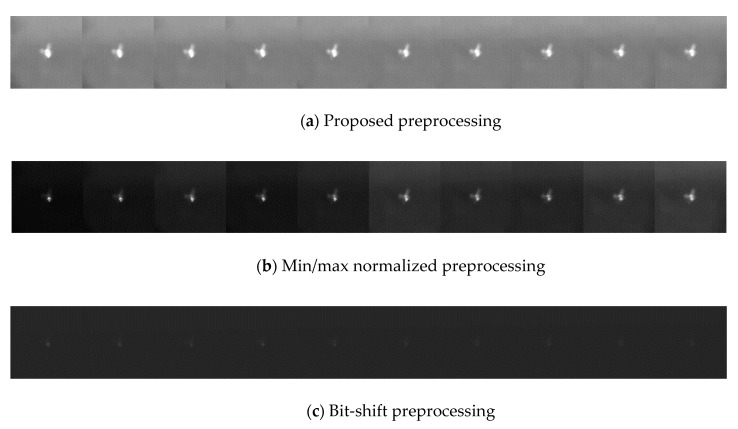
A comparison of the preprocessing results for consecutive frames. (**a**,**b**) display images that are converted to 8 bits via normalization. (**a**) uses the mean and standard deviation for cumulated images, while (**b**) uses the mean and standard deviation for the current image. The pixel distribution in (**a**) shows lower variation. The images in (**c**) employ the bit-shift method, leading to a loss of information.

First, two circular buffers with *N* frames are constructed to save the mean and standard deviation of the images, and these are updated in each buffer. Second, based on the current frame, the cumulated average of the mean and standard deviation are calculated with the most recent *N* frames (Equation (1) and Figure 2). This extracts the representative features from these recent images. Third, the min/max threshold values are decided using Equation (2). Finally, based on the threshold values, the current image can be converted to 8 bits using the normalizing operation in Equation (3). Equation (2) and Equation (3) lead to images with a lower variation in pixel intensity and a higher quality (Figure 3). If Equation (2) is not used, the pixel intensity of the images fluctuates dramatically between frames, making the image appear as if it is affected by illumination and thus more difficult to be trained on (Figure 3).

### 3.2. Training: Siamese-Based Deep Network with RPNs

VOT requires data with rich representations, such as various dimensions, scales, and resolutions. However, a single output layer for feature extraction is not sufficient even if the depth of features is rich in a CNN. Instead, combining several layers of feature extraction into one improves classification and localization.

**Figure 4:**
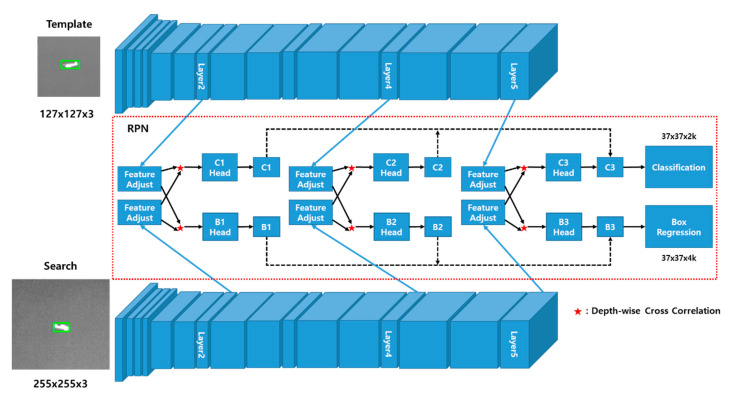
The proposed network architecture for the training phase. For feature extraction, a modified version of Siamese MobileNetV2 with a reduced number of layers is adopted. Region proposal networks (RPNs) lie between the Siamese networks. Each RPN has two branches, one for classification and the other for box regression, and they are formulated using depth-wise cross-correlation. As a consequence, the entire network outputs a dense prediction by fusing the three RPNs.

As shown in Figure 4 and Table 1, the proposed framework consists of a Siamese network for feature extraction and three RPNs for target classification and box regression. Specifically, MobilenetV2 [18] is adopted for its rich feature representations, though it is modified to simplify its structure. The three RPNs are fused to enable the robust tracking of a target location. As input patches, template and search images are fed into the proposed network and the entire network is trained end-to-end. 

#### 3.2.1. Siamese-Based Feature Extraction Network

The feature extraction network is depicted in Figure 4. The input to the network is a pair of patches cropped from the previous frame for the template and the current frame for tracking. To use various features with an identical patch size, the proposed feature extraction network consists of MobileNetV2, which has linear bottlenecks. However, adopting the existing MobileNetV2 wholescale is inefficient because the network does not operate in real-time. Therefore, we propose a modified network that is lighter than MobileNetV2 but that maintains the linear bottlenecks.

In order to simplify MobileNetV2, the number of existing bottlenecks is reduced from 7 to 5, and the number of repetitions for the bottlenecks is also reduced. In addition, the layers for classification that consist of two convolutions and average pooling are removed. However, the depth-wise separable convolutions, which are the key function of MobileNet [37], are adopted for all bottlenecks. Moreover, we add a receptive field using dilated convolutions [42] in the middle sequence of Layer 4 and Layer 5. The specifications for the feature extraction layers are presented in Table 1.

The proposed Siamese network consists of two branches: template, which receives the previous target patch as input and search, which receives the current target patch as input. They share all of the parameters in the CNN so that the two inputs are encoded by the same transformation. *z* denotes the input of the template branch and *x* denotes the input of the search branch. The output features of the Siamese network can thus be represented as $\left(\varphi_{2}\left(z\right),{\;\varphi }_{2}\left(x\right)\right),\;\left(\varphi_{4}\left(z\right),{\;\varphi }_{4}\left(x\right)\right),\;\mathrm{and}\;\left(\varphi_{5}\left(z\right),{\;\varphi }_{5}\left(x\right)\right)$. These multi-branch outputs are fed into the RPNs individually.

#### 3.2.2. Weighted Sum of the Region Proposal Networks

**Figure 5:**
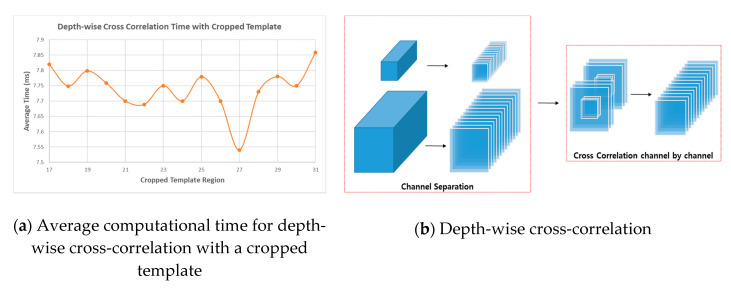
Description of depth-wise cross-correlation. (**a**) shows the computational time for cropped templates of various sizes. (**b**) displays a schematic summary of the depth-wise cross-correlation method. It predicts multi-channel correlation features between the template and search branches.

The RPNs consist of feature-adjustment, depth-wise cross-correlation, and result sections. The feature-adjustment sections are appended to each of the feature extraction outputs to balance the output channels. It also crops padding areas in the template branch to reduce computational time. As shown in Table 1, because 3 × 3 convolution sequences in all of the layers are retained as padding, the template and search features have 31 × 31 and 63 × 63 regions. Thus, it is burdensome to compute six cross-correlations. To overcome this, the padding area can be cropped from the features. However, search features cannot be cropped because they are placed in a certain position within a random shift range to prevent center bias overfitting. Therefore, the template features can be reduced up to a central 17 × 17 region, but we should optimize the time required for the cross-correlation between the 63 × 63 and cropped template. With this in mind, the most efficient method is to crop the template features to a central 27 × 27 region (Figure 5a). The depth-wise cross-correlation section consists of lightweight layers used to extract an efficient feature map. As shown in Figure 4, cross-correlation operations are computed six times, requiring high computational time. Inspired by depth-wise separable convolution [37], we adopt a depth-wise cross-correlation that is computed channel-by-channel (Figure 5b), significantly reducing the parameters and computational cost.

The result section has two branches, one for foreground and background classification and the other for box regression. If there are *k* anchors, the RPN outputs 2*k* channels for classification and 4*k* for regression. As mentioned above, the feature adjust section transforms φ(z) and φ(x) into $\varphi {\left(z\right)}_{adj}$ and $\varphi {\left(x\right)}_{adj}$ with the same number of channels using a 1 × 1 kernel. After the depth-wise cross-correlation with the $\varphi {\left(z\right)}_{adj}$ and $\varphi {\left(x\right)}_{adj}$, all of the outputs are calculated with 2*k* and 4*k* channels respectively using a 1 × 1 kernel, and we can get the results which consist of $C_{wxhx2k}^{cls}$ and $S_{wxhx4k}^{reg}$ (Equation (4)).

Thus, $\varphi {\left(z\right)}_{adj}$ serves as a correlation kernel filter for $\varphi {\left(x\right)}_{adj}$, meaning that depth-wise cross-correlation is computed for both the classification and box regression branches. Because each RPN, i.e., (C1, S1), (C2, S2), and (C3, S3) (Figure 4), has the same resolution, the weighted sum of each RPN is adopted as the final output (Equation (5)). The weight parameters in Equation (5) are optimized end-to-end with the network in the offline phase.

(4) $$\begin{array}{c} C_{wxhx2k}^{cls}=\varphi {\left(z\right)}_{adj}\;★\;\varphi {\left(x\right)}_{adj}\\ S_{wxhx4k}^{reg}=\varphi {\left(z\right)}_{adj}\;★\;\varphi {\left(x\right)}_{adj} \end{array}$$

(5) $$C_{all}^{cls}=\overset{5}{\underset{j=2}{\sum }}\alpha_{i}\times C_{j}^{cls}\;S_{all}^{reg}=\overset{5}{\underset{j=2}{\sum }}\beta_{i}\times S_{j}^{reg},\;\;j\neq 3$$

#### 3.2.3. Loss and Optimization Strategy

As shown in Figure 4, each $C_{all}^{cls}$ contains a 2*k* channel vector, which represents the positive and negative intersection of union (IOU) for each anchor. SoftMax is adopted to compute the classification branch for both the training and inference phases. Similarly, each $S_{all}^{reg}$ contains a 4*k* channel vector, which measures the normalized distance between the anchor and ground-truth. When the entire network is trained with *k* anchors, inspired by the loss in Faster R-CNN [19], we adopt cross-entropy loss and smooth L1 loss. They are employed for the classification branch and the box regression branch. Cross-entropy loss can be formulated as

(6) $$L_{cls}=-\frac{1}{\left|D\right|}\sum_{u\in D}y\left[u\right]\ln\left(v\left[u\right]\right)$$

where *v* is a real-valued classification map of the template-search pair and *y* is the positive and negative ground-truth IOU label. Using the proposed network, this produces the classification probability map v: D→ℝ.

Let (*A_x_*, *A_y_*, *A_w_*, *A_h_*) denote the center point and the size of the anchor box and let (*G_x_*, *G_y_*, *G_w_*, *G_h_*) denote the center point and the size of the ground-truth. The normalized distance *m* between the anchor box and the ground-truth is

(7) $$\begin{array}{c} m\left[0\right]=\frac{G_{x}-A_{x}}{A_{w}},\;m\left[1\right]=\frac{G_{y}-A_{y}}{A_{h}}\\ m\left[2\right]=\ln\frac{G_{w}}{A_{w}},\;m\left[3\right]=\ln\frac{G_{h}}{A_{h}} \end{array}$$

Smooth L1 loss is thus formulated as

(8) $$\begin{array}{c} L_{reg}=\overset{3}{\underset{i=0}{\sum }}\mathrm{SmoothL}1\left(m\left[i\right]\right)\\ \mathrm{SmoothL}1\left(x\right)=\{\begin{array}{c} 0.5x^{2},\;\;\left|x\right|<1\\ \left|x\right|-\frac{1}{2},\;\;\left|x\right|\geq 1 \end{array} \end{array}$$

Final loss can then be computed:

(9) $$L_{all}=\lambda_{1}L_{cls}+\lambda_{2}L_{reg}$$

where *λ*_1_ and *λ*_2_ are hyperparameters used to balance the two losses.

By using the combined loss, the main objective is to find the parameter *W* that minimizes the average loss of the proposed network. Assume the prediction function ζ(x;W), which has *n*-sample search *x_i_* and corresponding label *ℓ_i_*_._ The average loss that minimizes parameter *W* is

(10) $$arg\;\underset{W}{min}\frac{1}{n}\overset{n}{\underset{i=1}{\sum }}L\left(\zeta \left(x_{i};W\right),\;\ell_{i}\right).$$

The parameter *W* of the prediction function can be learned from the *n*-sample template *z_i_* using the feed-forward function *Φ*, which maps $\left(z;W^{\prime }\right)$ to *W*:

(11) $$arg\;\underset{W^{\prime }}{min}\frac{1}{n}\overset{n}{\underset{i=1}{\sum }}L\left(\zeta \left(x_{i};\phi \left(z_{i};W^{\prime }\right)\right),\;\ell_{i}\right)$$

The prediction function ζ with parameter *W* consists of the Siamese feature extraction function as φ and the RPN function as ω. Therefore, Equation (11) can be reformulated using φ, ω, and *W* of the proposed network for the problem:

(12) $$arg\;\underset{W}{min}\frac{1}{n}\overset{n}{\underset{i=1}{\sum }}L\left(\omega \left(\varphi \left(x_{i};W\right);\varphi \left(z_{i};W\right)\right),\;\ell_{i}\right)$$

As seen in Equation (12), $\varphi \left(z_{i};W\right)$ is the information for the class of interest for finding the target of search patch *x*. In this way, the template branch, $\varphi \left(z_{i};W\right)$, can be reinterpreted as the training parameters for the kernel filter. In other words, if the network is trained properly in the training phase and the template branch in the inference phase has rich information from the past to the current frame, it can robustly track any arbitrary object. Therefore, the template branch embeds target information into the kernel filter and the search branch predicts the target location using this information.

### 3.3. Inference Process

#### 3.3.1. Kernel Filter

**Figure 6:**
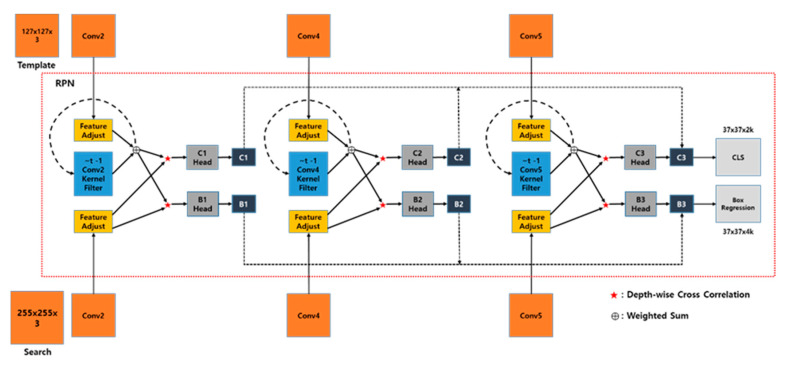
Proposed network architecture for the inference phase. Before depth-wise cross-correlation, the kernel filter in the template branch is updated to store the information from the past to the current feature. Adopting the weighted kernel filter improves tracking performance.

As mentioned in Section 3.2.3, the Siamese template branch $\varphi \left(z_{i};W\right)$ functions as the kernel filter. Therefore, tracking performance can be improved if the kernel filter has the information from the past and the current feature. For this reason, we propose a kernel filter with weighted summation. As shown in Figure 6, the weighted kernel filters are summed with the current feature and then updated using a loop-back method. The weighted kernel filter is formulated as

(13) $$kernel_{t}=\{\begin{array}{c} \varphi {\left(z_{t}\right)}_{adj},\;\;\;\;\;\;\;\;\;\;\;\;\;\;t=0\\ \left(1-\eta \right)\times kernel_{t-1}+\eta \times \varphi {\left(z_{t}\right)}_{adj},\;otherwise \end{array}$$

where *t* represents the frame index and *ƞ* is the online learning rate.

#### 3.3.2. Box Decoding and Selection

As shown in Equation (5), the RPNs conduct online inference with the weighted kernel filter (Equation (13) and Figure 6). A forward pass on the proposed network is conducted to obtain the classification and the box regression outputs. Given the notation defined in Equation (5), the classification and box regression feature map are given as

(14) $$C_{all\left(w\times h\times 2k\right)}^{cls}=I_{i,\;j,\;m}^{cls}$$

where 0≤i<w, 0≤j<h, 0≤m<2k

(15) $$S_{all\left(w\times h\times 4k\right)}^{reg}=\left\{\left(dx_{i,\;\;j,\;n}^{reg},\;dy_{i,j,\;n}^{reg},dw_{i,\;j,n}^{reg},dh_{i,\;j,n}^{reg}\right)\right\}$$

where 0≤i<w, 0≤j<h, 0≤n<k.

Because variables *i* and *j* encode the location of the corresponding anchor, and *m* encodes the ratio of the corresponding anchor, the corresponding anchor set can be derived as $\left\{\left(x_{i}^{an},\;y_{j}^{an},w_{m}^{an},h_{m}^{an}\right)\right\}$. Therefore, $S_{all\left(w\times h\times 4k\right)}^{reg}$ can be decoded to the set $RES=\left\{\left(dx_{i},dy_{j},dw_{l},dh_{l}\right)\right\}$ using the anchor set:

(16) $$\begin{array}{c} dx_{i}=dx_{i,\;\;j,\;n}^{reg}\times w_{m}^{an}+x_{i}^{an}\\ dy_{j}=dy_{i,j,\;n}^{reg}\times h_{m}^{an}+y_{i}^{an}\\ dw_{l}=exp\left(dw_{i,\;j,n}^{reg}\right)\times w_{m}^{an}\\ dh_{l}=exp\left(dh_{i,\;j,n}^{reg}\right)\times h_{m}^{an}. \end{array}$$

After decoding the box regression (Equation (16)), a scale penalty and cosine window are adopted to re-rank the decoded box information and determine the best score. The cosine window is added to prevent significant displacement from the previous target and the scale penalty is added to prevent a large change in the scale and ratio. The scale penalty is represented as

(17) $$penalty=exp\left(-\left(max\left(\frac{r}{r^{\prime }},\frac{r^{\prime }}{r}\right)\times max\left(\frac{s}{s^{\prime }},\frac{s^{\prime }}{s}\;\right)-1\right)\times k\right)$$

where *k* is a hyperparameter, *r* represents the previous target ratio for the width and height, and *r*’ represents the ratio of *RES*. *s* represents the previous target scale for the width and height and *s*’ represents the scale of *RES*. They can be expressed as

(18) $$r=\frac{w}{h},\;\;s=\sqrt{\left(w+pad\right)\times \left(h+pad\right)}$$

where *pad* is equal to $\frac{w+h}{2}$.

Afterwards, the final classification output is re-ranked and the best score chosen. Finally, the target information, including the bounding box and center points, is smoothly updated.

## 4. Experimental Results

This section provides the details of the proposed method and evaluates the proposed tracking algorithm by comparing it with other trackers using the anti-air TIR dataset described in Section 3.1. The effectiveness of the proposed network is evaluated by modifying the number of the feature extraction output layers in ablation analysis and by running the network with and without the weighted kernel filter described in Section 3.3.1. Finally, qualitative results for the anti-air TIR test set are presented.

### 4.1. Implementation Details

As previously discussed, this study uses a modified version of MobileNetV2 that does not employ existing pre-trained parameters because the domain is different. Therefore, the entire network is trained end-to-end using the anti-air TIR dataset. All of the parameters are trained by optimizing the combined loss in Equation (9) with stochastic gradient descent (SGD) using one GPU. A total of 30 epochs are run with a batch size of 32, which takes about 60 h to converge. During the first seven epochs, the learning rate is fixed at 0.001 for the RPNs and 0.005 for the backbone network. For the remaining 23 epochs, the learning rate for the entire network is decreased in log space from 0.005 to 0.0005. The input image pairs from the anti-air TIR dataset are extracted with an interval of fewer than 10 frames and then cropped. If the bounding box of the target is denoted as (w, h), the input patch is cropped around the center with a size of A×A. The equation is the same as the value for *s* in Equation (18). The target is now placed in the center of the area A×A and then the target position is randomly shifted within A×A. Afterwards, it is resized to 255×255 for the search and 127×127 for the template. During the inference phase, the kernel filter of the template branch and the target position are continuously updated. The experiments are implemented using PyTorch on Ubuntu 16.04 with an Intel i7 processor (Intel Co., Santa Clara, CA, USA), 32 GB of RAM (Samsung Co., Suwon, Korea), and an Nvidia RTX 2080Ti (Nvidia Co., Santa Clara, CA, USA).

### 4.2. Ablation Analysis

In order to analyze the effectiveness of the proposed method, ablation analysis is conducted on the anti-air TIR test set. In total, ten experiments are conducted (Table 2, Table 3 and Table 4). We also analyzed the affection of the preprocessing method described in Section 3.1.2.

#### 4.2.1. Ablation Analysis on Selecting the Feature Extraction Layers

**Figure 7:**
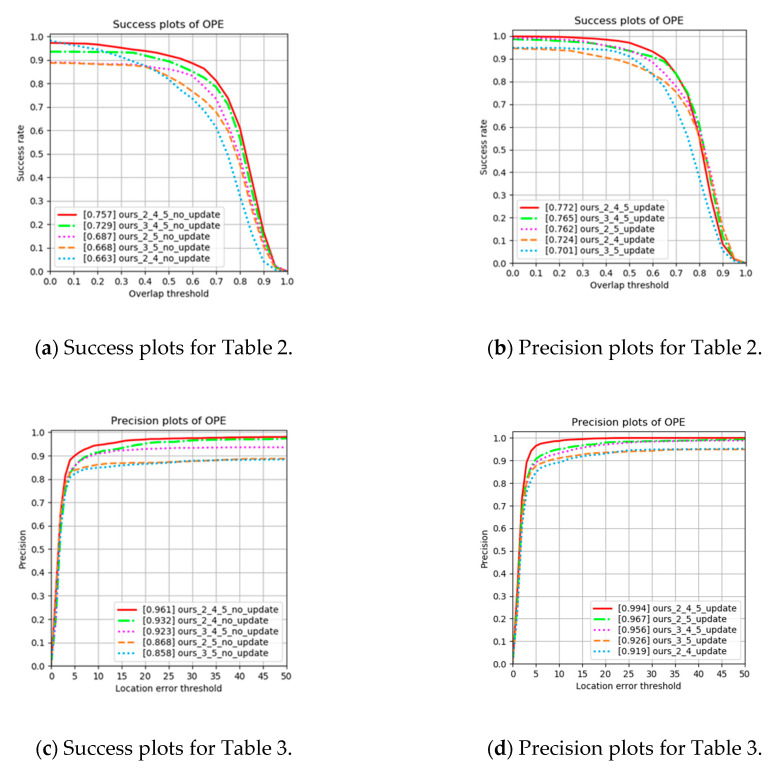
(**a**,**c**) Success and (**b**,**d**) precision plots for Table 2 and Table 3 on ablation analysis.

The choice of feature-extraction layers is important because the number of parameters directly affects the speed and performance of the tracker. Therefore, we select layers that are able to be run in real-time. When using two layers, consecutive layers are avoided as the purpose of feature extraction is to use appearance and semantic features. Based on the information presented in Table 2, Table 3, and Figure 7, three feature-extraction layers (No 4. and No. 5 in Table 2 and Table 3) were found to produce better results than two feature-extraction layers. Also, using layers 2, 4, and 5, which is our proposed feature extraction method, produces the best result.

#### 4.2.2. Ablation Analysis on Adopting the Weighted Kernel Filters

**Figure 8:**
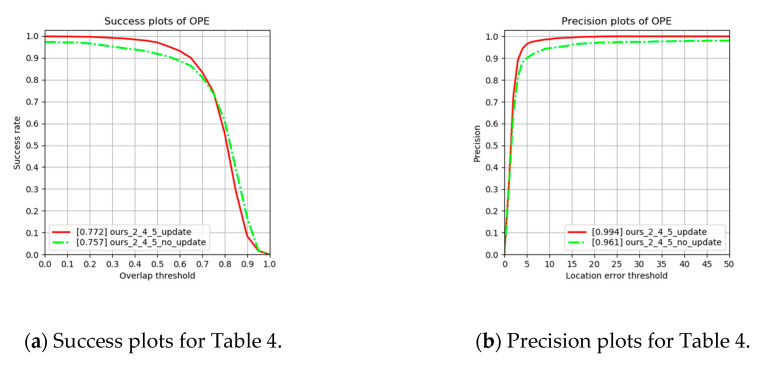
(**a**) Success and (**b**) precision plots for Table 4 on ablation analysis.

As shown in Section 4.2.1, the proposed backbone network is found to produce the best result. Therefore, we remain the proposed backbone network and compare the impact of the weighted kernel filter. As shown in Table 4 and Figure 8, the adoption of the weighted kernel filter improves the success rate. This is because balanced template branches, which have various information from the past to the current feature, are embedded in the filter, stabilizing the inference process.

Overall, adopting the proposed backbone network and the weighted kernel filter produces the best performance in terms of success and precision plots.

#### 4.2.3. Ablation Analysis on Adopting the Preprocessing Method

**Figure 9:**
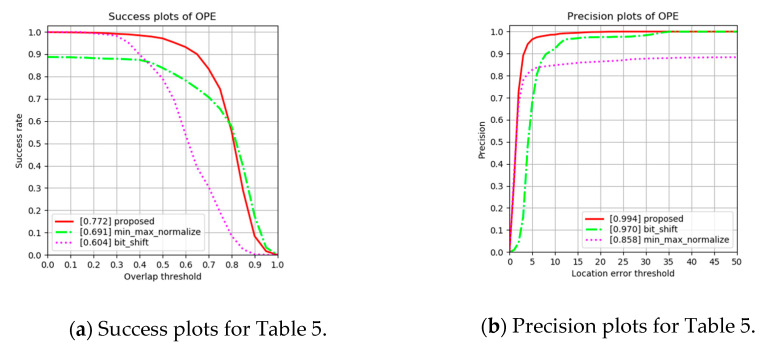
(**a**) Success and (**b**) precision plots for Table 5 on ablation analysis.

The choice of the preprocessing method affects the tracking performance. For the ablation analysis, we remain the proposed backbone network and kernel filter. As shown in Table 5 and Figure 9, the proposed preprocessing method is the best tracking performance. This is because less variation of image intensity stabilizes the training process and this leads to facilitation of the inference process.

### 4.3. Evaluation Methodology

The proposed tracking method is compared with six state-of-the-art trackers: DSST [5], DCFNet [9], ECO [10], SiamFC [11], SiamRPN [15], and Sa-Siam [43]. For extensive experimental validation [44], one-pass evaluation (OPE) is employed with success and precision plots. The success plots measure the overlap ratio using the IOU between the ground-truth and the estimated bounding boxes. The precision plots measure the variation between the ground-truth and the estimated center points. They both plot the percentage under the threshold values. The distance threshold is set to 20 for the precision plots, and area under the curve is used for the success plots.

### 4.4. Evaluation Using the Anti-Air TIR Dataset

**Figure 10:**
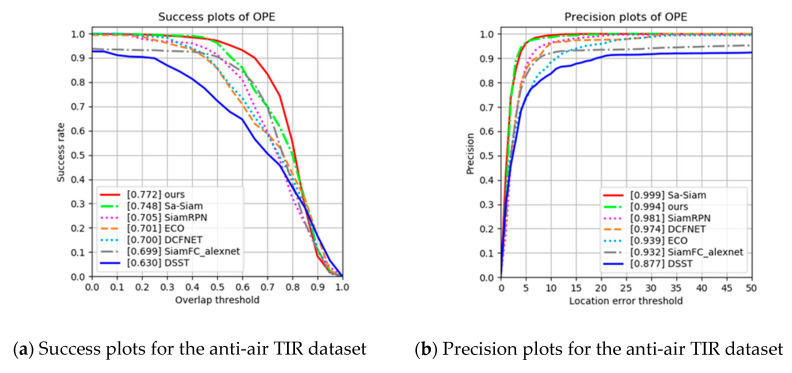
(**a**) Success and (**b**) precision plots for one-pass evaluation (OPE) using the anti-air TIR dataset.

As described in Section 3.1, data is collected from TIR cameras, leading to 78,903 training images, 21,046 validation images, and 14,750 test images. The proposed tracking method is evaluated using the test images with OPE and the overlap ratio success and distance precision. Compared with the state-of-the-art trackers, Figure 10 shows the proposed tracker is the highest ranked in the success plots. In particular, compared with SiamRPN [15], which is a baseline tracker, the proposed tracker has a 6.7% higher overlap ratio and a 1.3% higher precision ratio.

### 4.5. Qualitative Evaluation

**Figure 11:**
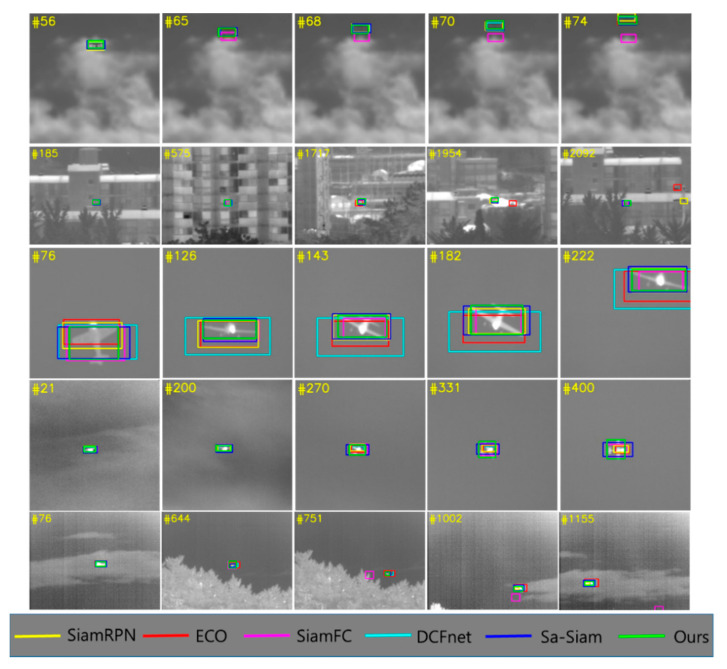
Qualitative results for our tracker and five comparison trackers using the anti-air TIR test set. The proposed tracker achieves the best performance for several challenging sequences.

Qualitative testing of the proposed tracker and five other trackers (SiamRPN, SiamFC, DCFNet, ECO, and Sa-Siam) is conducted. Figure 11 presents several frames from five challenging sequences from the anti-air TIR test set. In the first and second columns, our tracker can robustly track the target even when it merges with the background and its shape disappears. In the third and fourth columns, the proposed tracker can accurately track the object even with sudden positional changes while other trackers struggle to estimate the bounding boxes. As shown in the last column, the target exhibits significant scale variation by rotating nearly 180 degrees. Nevertheless, our tracker can still more accurately estimate the bounding boxes than the other trackers. In particular, our method shows a significant improvement compared with the baseline tracker SiamRPN.

## 5. Conclusions

In this paper, a deep-learning-based anti-air TIR object tracking method that employs its own TIR dataset was proposed. The dataset was created by adopting a preprocessing procedure with circular queue buffers using the mean and standard variation of the images. The proposed algorithm consists of Siamese-based feature extraction using a modified version of MobileNetV2 and the weighted summation of RPNs. It outputs classification and box regression, and combined loss is adopted using cross-entropy and smooth L1. Compared with prominent state-of-the-art trackers, the proposed tracker was shown to robustly estimate the bounding box position with higher accuracy. The proposed method will help researchers to further develop deep-learning-based IR trackers for electro-optical systems.

## Figures and Tables

**Table 1:** Details of the proposed feature extraction network. Each column describes a sequence of 1 or more modulo stride layers, repeated *n* times. All layers have *c* output channels. The first sequence of all layers has stride *s*. All of the convolutions, except for channel expansion and reduction, use a 3 × 3 kernel. The input channels for each sequence are multiplied by the expansion factor *t*.

Template Input	Search Input	Operator	*t*	c	*n*	s	Remark
$127^{2}\times 3$	$255^{2}\times 3$	conv2D	-	32	1	2	Layer 0
$63^{2}\times 32$	$127^{2}\times 32$	bottleneck	1	16	1	1	Layer 1
$63^{2}\times 16$	$127^{2}\times 16$	bottleneck	6	24	2	2	Layer 2
$31^{2}\times 24$	$63^{2}\times 24$	bottleneck	6	32	3	1	Layer 3
$31^{2}\times 32$	$63^{2}\times 32$	bottleneck	6	64	3	1	Layer 4
$31^{2}\times 64$	$63^{2}\times 64$	bottleneck	6	160	1	1	Layer 5
$31^{2}\times 160$	$63^{2}\times 160$	-	-	-	-	-	-

**Table 2:** Ablation analysis for the number of feature layers without the weighted kernel filter on anti-air TIR test set. Considering real-time speed, using all layers is excluded.

No.	Layer 2	Layer 3	Layer 4	Layer 5	Weighted Kernel Filter	Overlap Ratio
1	O	X	O	X	X	0.663
2	O	X	X	O	X	0.687
3	X	O	X	O	X	0.668
4	X	O	O	O	X	0.729
5	O	X	O	O	X	0.757

**Table 3:** Ablation analysis for the number of feature layers with the weighted kernel filter on anti-air TIR test set. Considering real-time speed, using all layers is excluded.

No.	Layer 2	Layer 3	Layer 4	Layer 5	Weighted Kernel Filter	Overlap Ratio
1	O	X	O	X	O	0.724
2	O	X	X	O	O	0.762
3	X	O	X	O	O	0.701
4	X	O	O	O	O	0.765
5	O	X	O	O	O	0.772

**Table 4:** Ablation analysis for the weighted kernel filter on the anti-air TIR test set. We use the proposed backbone network as it produces the best result. The weighted kernel filter updates the template branch from the past to the current feature if it is used.

No.	Layer 2	Layer 3	Layer 4	Layer 5	Weighted Kernel Filter	Overlap Ratio
1	O	X	O	O	X	0.757
2	O	X	O	O	O	0.772

**Table 5:** Ablation analysis for selecting the preprocessing method on the anti-air TIR test set. As described Section 3.2.1, three preprocessing methods are adopted.

No.	Preprocessing Method	Overlap Ratio
1	Proposed	0.772
2	Min/Max Normalized	0.691
3	Bit-shift	0.604
